# Fundamental considerations for the use of hyaluronidase, an enzyme for degrading HA fillers

**DOI:** 10.1111/srt.13839

**Published:** 2024-07-01

**Authors:** Gi‐Woong Hong, Kyu‐Ho Yi

**Affiliations:** ^1^ Sam Skin Plastic Surgery Clinic Seoul South Korea; ^2^ Division in Anatomy and Developmental Biology, Department of Oral Biology, Human Identification Research Institute, BK21 FOUR Project Yonsei University College of Dentistry Seoul South Korea; ^3^ Maylin Clinic (Apgujeong) Seoul South Korea

Dear Editor,

HA fillers can be dissolved using the enzyme hyaluronidase, which can address complications or dissatisfaction arising from HA filler injections. Hyaluronidase is particularly useful in urgent situations where vascular complications have occurred due to intravascular HA filler injection.

Instances, where hyaluronidase might be injected post‐HA filler procedure, include cases where the filler was not injected in the desired location, excessive amounts were injected, significant asymmetry is present, the injected filler has migrated, the filler has formed hard lumps or beads, discomfort is experienced during facial movements due to the injected filler mass, patient dissatisfaction with the appearance, swelling, and inflammation around the filler due to hypersensitivity, severe foreign body reactions forming granulomas, Tyndall effect causing bluish discoloration of the skin, and suspected or confirmed vascular complications due to the filler (Table 1).

**TABLE 1:** Fundamental considerations for the use of hyaluronidase.

Consideration	Description
Purpose	Hyaluronidase is used to dissolve HA fillers, especially in cases of incorrect injection, excessive filler, asymmetry, migration, lumps, discomfort, patient dissatisfaction, inflammation, granulomas, Tyndall effect, and vascular complications.
Dosage	Historically, 5‐15 units of hyaluronidase dissolve 0.1 mL of HA filler, but for modern fillers with stronger cross‐linking, 200‐300 units per 1 mL for wrinkles and over 500 units per 1 mL for volumizing fillers may be necessary (Figure 1).
Time to effect	The reaction time varies for biphasic and monophasic fillers. Understanding these times is crucial for effective emergency response to vascular complications.
Duration of activity	Hyaluronidase retains functionality for approximately 6 h post‐injection. In cases of persistent filler masses, repeated injections may be needed after a day. Hyaluronidase exposed to blood within vessels loses activity rapidly, often within minutes, requiring quick and repeated administration.
Approach for vascular complications	Larger quantities (1500‐3000 units) of hyaluronidase may be needed, injected widely around the ischemic area to facilitate vascular infiltration. Follow‐up injections may be necessary to restore blood flow (Figure 2).
Dilution and injection technique	Hyaluronidase should be diluted in sufficient saline and massaged into the injection site for even distribution and maximum contact with the HA filler. Given its large molecular size, it acts on particles it directly contacts, increasing surface area exposure gradually (Figure 3).
Injection into filler masses	For lumps or nodules with poor blood circulation and thicker collagen capsules, hyaluronidase should be injected directly into the filler mass to effectively penetrate between particles.
Allergic reactions	Be prepared for potential allergic reactions such as itching and redness. Conduct a skin test beforehand if necessary, and have antihistamines and steroids available for treatment.

First, consider the dosage of hyaluronidase required to dissolve HA fillers, measured in units. Historically, it was suggested that 5–15 units of hyaluronidase are sufficient to dissolve 0.1 mL of HA filler, making 150 units enough for 1 mL of HA filler (Figure 1). However, this quantity may be insufficient for modern HA fillers designed with stronger cross‐linking to enhance volume. For softer HA fillers used for wrinkles, a minimum of 200–300 units per 1 mL is typically required, while for volumizing fillers, over 500 units per 1 mL may be necessary.^1^, ^2^, ^3^

**FIGURE 1:**
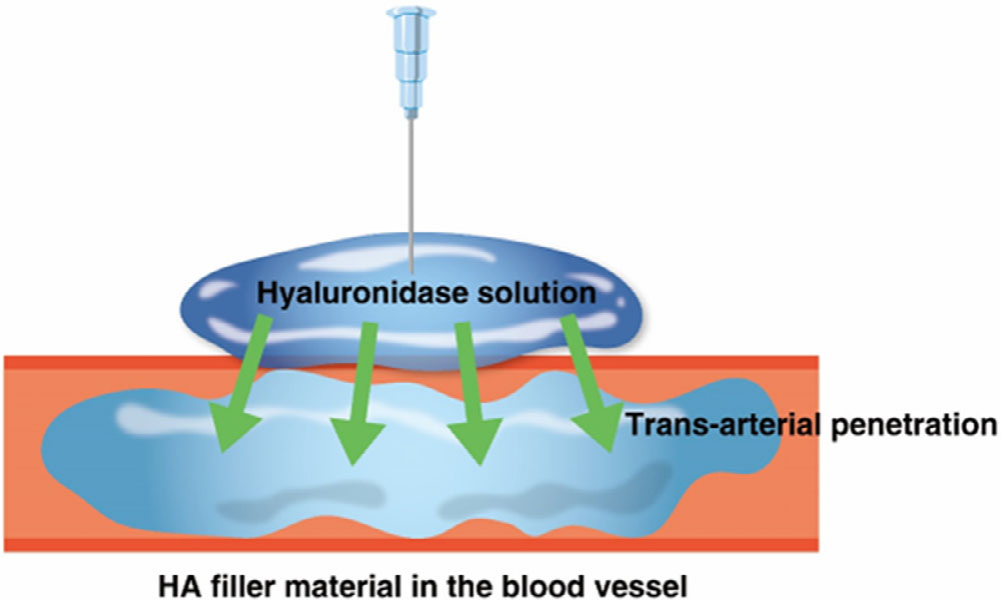
Infiltration of hyaluronidase solution through the vessel wall.

Second, the time required for hyaluronidase to take effect should be anticipated. Understanding the reaction times for biphasic and monophasic fillers to hyaluronidase is crucial, especially in emergency situations involving vascular complications, to ensure optimal use of hyaluronidase for effective results.

Third, consider the duration of hyaluronidase activity post‐injection. Studies indicate that hyaluronidase retains functionality for approximately 6 h after injection. Repeated injections might be needed for persistent HA filler masses, typically administered after a day. Hyaluronidase exposed to blood within vessels loses its enzymatic activity rapidly, often within minutes, necessitating quick and possibly repeated administration.^2^

Fourth, when injecting hyaluronidase to address vascular complications, a different approach is needed compared to dissolving filler masses. A larger quantity of hyaluronidase may be required, up to 1500–3000 units, and it should be injected widely around the ischemic area to facilitate vascular infiltration. Follow‐up injections may be necessary to restore blood flow.^4^, ^5^, ^6^

Fifth, hyaluronidase should be diluted in sufficient saline and massaged into the injection site to ensure even distribution and maximize contact with the HA filler. Given hyaluronidase's large molecular size, it cannot penetrate filler particles but acts on those it directly contacts, gradually increasing surface area exposure (Figure 2).^7^, ^8^, ^9^

**FIGURE 2:**
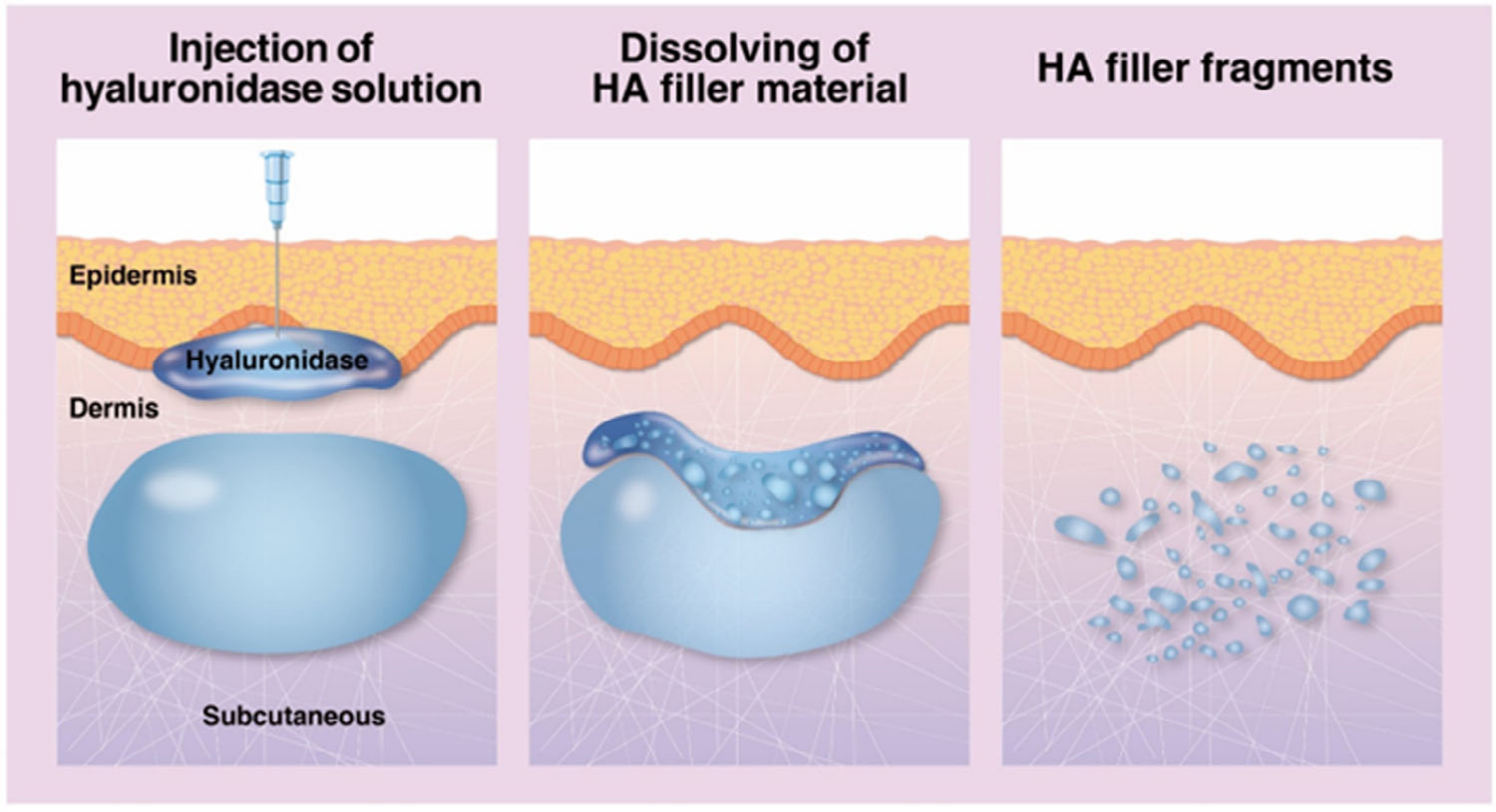
The action mechanism of hyaluronidase acting on HA filler material.

Sixth, filler masses forming lumps or nodules often have poor blood circulation and thicker collagen capsules, impeding effective hyaluronidase action. In such cases, hyaluronidase should be injected directly into the filler mass to penetrate between the particles effectively (Figure 3).^10^

**FIGURE 3:**
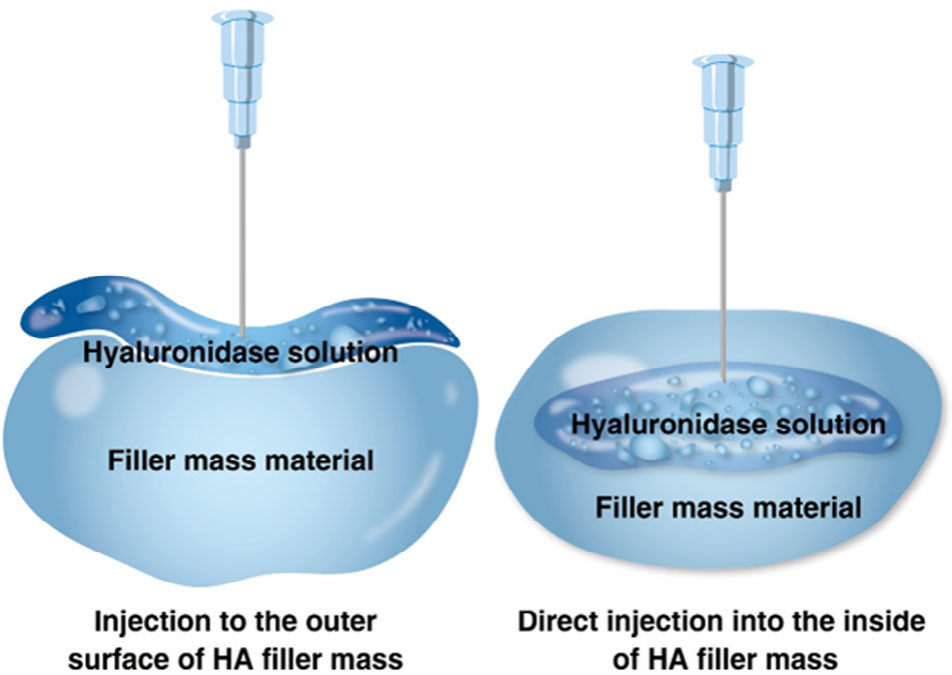
Two injection methods of hyaluronidase solution for HA filler mass.

Last, since hyaluronidase injections can trigger allergic reactions such as itching and redness, it is essential to be prepared for such occurrences, potentially conducting a skin test beforehand and having antihistamines and steroids available for treatment.

## CONFLICT OF INTEREST STATEMENT

We acknowledge that we have considered the conflict‐of‐interest statement included in the “Author Guidelines.” We hereby certify that, to the best of our knowledge, no aspect of our current personal or professional situations might reasonably be expected to significantly affect our views on the subject we are presenting.

## Data Availability

Research data are not shared.
